# Mincle receptor in macrophage and neutrophil contributes to the unresolved inflammation during the transition from acute kidney injury to chronic kidney disease

**DOI:** 10.3389/fimmu.2024.1385696

**Published:** 2024-05-17

**Authors:** Cui Wang, Yilin Zhang, Anran Shen, Taotao Tang, Ning Li, Chuanhui Xu, Bicheng Liu, Linli Lv

**Affiliations:** Institute of Nephrology, Zhongda Hospital, Southeast University School of Medicine, Nanjing, Jiangsu, China

**Keywords:** unresolved inflammation, Mincle, macrophage, neutrophil, acute kidney injury, chronic kidney disease

## Abstract

**Background:**

Recent studies have demonstrated a strong association between acute kidney injury (AKI) and chronic kidney disease (CKD), while the unresolved inflammation is believed to be a driving force for this chronic transition process. As a transmembrane pattern recognition receptor, Mincle (macrophage-inducible C-type lectin, Clec4e) was identified to participate in the early immune response after AKI. However, the impact of Mincle on the chronic transition of AKI remains largely unclear.

**Methods:**

We performed single-cell RNA sequencing (scRNA-seq) with the unilateral ischemia-reperfusion (UIR) murine model of AKI at days 1, 3, 14 and 28 after injury. Potential effects and mechanism of Mincle on renal inflammation and fibrosis were further validated *in vivo* utilizing Mincle knockout mice.

**Results:**

The dynamic expression of Mincle in macrophages and neutrophils throughout the transition from AKI to CKD was observed. For both cell types, Mincle expression was significantly up-regulated on day 1 following AKI, with a second rise observed on day 14. Notably, we identified distinct subclusters of Mincle^high^ neutrophils and Mincle^high^ macrophages that exhibited time-dependent influx with dual peaks characterized with remarkable pro-inflammatory and pro-fibrotic functions. Moreover, we identified that Mincle^high^ neutrophils represented an “aged” mature neutrophil subset derived from the “fresh” mature neutrophil cluster in kidney. Additionally, we observed a synergistic mechanism whereby Mincle-expressing macrophages and neutrophils sustained renal inflammation by tumor necrosis factor (TNF) production. Mincle-deficient mice exhibited reduced renal injury and fibrosis following AKI.

**Conclusion:**

The present findings have unveiled combined persistence of Mincle^high^ neutrophils and macrophages during AKI-to-CKD transition, contributing to unresolved inflammation followed by fibrosis via TNF-α as a central pro-inflammatory cytokine. Targeting Mincle may offer a novel therapeutic strategy for preventing the transition from AKI to CKD.

## Introduction

As a widespread clinical syndrome with acute renal dysfunction, acute kidney injury (AKI) could progress to chronic kidney disease (CKD) with high risk, which is characterized with fibrotic structural lesions (1). However, the mechanism underlies the progression from AKI to CKD remains unclear. Inflammation plays a dual role, being essential for renal repair while also acting as a potential driver of sustained kidney damage leading to CKD (2–5). Therefore, depicting the mechanism of unresolved inflammation during this chronic transition process is of great significance.

The onset of AKI prompts an immediate and robust innate inflammatory response characterized by rapid recruitment of neutrophils and natural killer cells followed by infiltration and activation of monocytes/macrophages and resident dendritic cells, which further stimulate the adaptive immune responses (3, 6). The innate immunity triggered by AKI produce a spectrum of inflammatory mediators, meanwhile, damage-associated molecular patterns (DAMPs) facilitate the recruitment and/or persistence of various inflammatory cells (3, 7). As the disease progresses, if injury is not resolved, prolonged inflammation can result in maladaptive repair and subsequent uncontrolled fibrosis (8–10). However, the precise mechanism driving the formation of the inflammatory milieu and the role of innate immune cells in the unresolved situation have yet to be completely delineated. Phenotypic and functional plasticity of innate immune cells increase the complexity of the context. Recently, emerging single-cell genomic studies have provided novel insights into the heterogeneity of innate immune cells during the process of renal inflammation and fibrosis (11–14).

Mincle (macrophage-inducible C-type lectin, Clec4e), recognized as a pattern recognition receptor is mainly expressed in innate immune cells including monocytes/macrophages, neutrophils and dendritic cells (15–17). Previous researches have uncovered the significant involvement of Mincle in both infectious diseases and sterile inflammation, wherein it prompts the secretion of pro-inflammatory cytokines and chemokines upon activation by its ligands encompassing pathogen-associated molecular patterns (PAMPs) or DAMPs (18–20). We have previously reported that Mincle could induce aggravated renal inflammation in the context of AKI by maintaining pro-inflammatory phenotype of macrophages, thereby contributing to the deterioration of kidney injury (21, 22). Importantly, Mincle was suggested to recognize β-glucosylceramide and free cholesterol released from dead tubular cells, thereby contributing to the cell death-induced sustained inflammation and renal atrophy (23). However, given the diversity and heterogeneity of innate immune cells, the dynamic profiling of Mincle in distinct populations of these cells and their contribution to the chronic progression of AKI after the initial kidney injury remains poorly understood.

Here, we applied single-cell RNA sequencing (scRNA-seq) and spatial transcriptomics in a unilateral ischemia-reperfusion (UIR) murine model of AKI at days 1, 3, 14 and 28 after injury. This study unveiled the combined persistence of Mincle^high^ neutrophils and macrophages during AKI-to-CKD transition, contributing to unresolved inflammation followed by fibrosis via tumor necrosis factor (TNF) as a central pro-inflammatory cytokine. Mincle-deficient mice showed improved renal pathology and reduced extracellular matrix deposition, suggesting that targeting Mincle may offer a novel therapeutic avenue for halting chronic progression of AKI.

## Materials and methods

### Preparation of single-cell suspension

Each kidney sample from three mice was minced and digested using the Multi Tissue dissociation kit (Miltenyi, 130-110-203), followed by homogenization through syringe-based mechanical disruption. The kidney tissue was enzymatically digested using a mixture of collagenase I, collagenase IV, and hyaluronidase in 1640 medium (Gibco, USA) at 37°C for 40 minutes while suppressing the response with 10% fetal bovine serum (FBS). The resulting dissociated solution was passed through 70-μm cell strainer and then was centrifuged at 400g for 5min at 4°C to collect the cell pellet. To remove any remaining erythrocytes, the red blood cell (RBC) lysis solution (Miltenyi,130-094-183) was applied on ice. Finally, the single-cell suspension was obtained with over 90% viability as detected by Countstar (Alit Biotech, Rigel S2).

### Single-cell RNA-seq library generation and sequencing

This process was performed by CapitalBio Technology in Beijing. The harvested cell suspension was processed with the Chromium single-cell controller (10x Genomics, GCG-SR-1) and the Single Cell G Chip Kit (10x Genomics, 1000120) to generate the single-cell gel beads in the emulsion. The reverse transcription was performed with the S1000TM Touch Thermal Cycler (Bio Rad) following a procedure of 53°C for 45 minutes, 85°C for 5 minutes and a subsequent hold at 4°C. The official library kit (Single Cell 3’ Library and Gel Bead Kit V3.1) was applied to construct the libraries intended for single-cell RNA-seq analysis. After the cDNA synthesis and amplification, the cDNA quality assessment was analyzed using Agilent 4200 instrument, and then sequenced on an Illumina Novaseq 6000 sequencer with paired-end reads of length 150 bp. The minimum sequencing depth per cell required was set at 100,000 reads.

### Analysis of single-cell RNA-seq data

#### Alignment and quality control

The cleaned raw FASTQ files underwent alignment to the mm10 (Ensembl GRCm38.93) reference genome and quantification using CellRanger (Version 6.0). Following data quality control, preprocessing, and dimensional reduction analysis performed by Seurat, a merged gene-cell data matrix was generated from all 15 matrices, comprising 12 UIR samples and 3 control samples. Prior to downstream analysis, low-quality cells with fewer than 200 expressed genes or mitochondrial gene percentages exceeding 25% were excluded. The remaining high-quality cell barcodes were exported.

#### Identification of marker genes and differentially expressed genes (DEGs)

For subsequent analysis, the remaining 60,010 high-quality single cells underwent a repeated Seurat process to generate the final dataset. The identification of DEGs in cell clusters was performed using the FindAllMarkers function implemented in the Seurat package. A comprehensive list of cell markers was employed for cell type annotation of all identified clusters in the final dataset.

#### Cell sub-clustering analysis

For cell sub-clustering, the entire Seurat pipeline was re-executed with identical parameters only in the barcodes of cells labeled as monocyte/macrophage and neutrophil. As a result, a total of 7 distinct subclusters of the monocyte/macrophage populations and 3 distinct subclusters of the neutrophil populations were identified.

#### Enrichment analysis

The GO (gene ontology) pathway enrichment analysis was conducted by the KOBAS software incorporating the Benjamini-Hochberg multiple testing adjustment. The top 50 DEGs (**Supplementary Table S1**) of each cluster were used as input for the enrichment. The obtained results were visualized using the R package.

#### The scoring of gene sets in scRNA-seq data

Gene sets comprising relevant markers were collected from previously relevant literatures in combination with GO database (**Supplementary Table S2**). The Seurat package’s “AddModuleScore” function was used for gene set scores of each cell cluster.

#### Cell trajectory analysis

The inference of cell developmental trajectory was conducted using RNA velocity according to the instructions (24). The state of mRNA over time can be inferred by RNA Velocity through the analysis of dynamic changes in alternative splicing of mRNA. Specifically, by incorporating both spliced and unspliced data, we employed the Python-based Velocyto command-line tool and the Velocyto.R package to calculate the RNA velocity and visualize it on the uniform manifold approximation and projection (UMAP) graph.

#### Ligand–receptor interaction analysis

The CellChat library was utilized to analyze cell-to-cell communication based on single-cell transcriptome data, enabling an investigation into inter-cellular cross-talk among diverse cell types (25). To forecast ligand-receptor interactions specific to each cell type, we employed the Python package CellChat with database v1.1.3. We considered only receptors and ligands expressed in over 5% of cells for analysis and visualization.

### Spatial transcriptome sequencing

This process was performed by CapitalBio Technology in Beijing. The kidney cryosections (10μm thickness) were carefully positioned on the Thermocycler Adaptor with the active surface facing upwards and incubated at 37°C for 1 minute. Subsequently, they were fixed in -20°C methyl alcohol for 30 minutes. The Visum spatial gene expression slide and Reagent Kit (10x Genomics, PN-1000184) were utilized for processing the Visum spatial gene expression analysis. A volume of 70 μl of permeabilization enzyme was introduced and incubated at 37°C for 30 minutes. A total volume of 100 μl of SSC was used to rinse each well, after which 75 μl of reverse transcription Master Mix was added for cDNA synthesis. Upon completion of first-strand synthesis, the RT Master Mix was removed from the wells. A subsequent step involved incubating each well with 75 μl of a solution containing 0.08 M KOH at room temperature for 5 minutes, followed by removal of KOH and washing with EB buffer (100 μl). In the process of second-strand synthesis, each well received an addition of Second Strand Mix (75 μl). The cDNA amplification procedure was conducted using a Bio Rad S1000TM Touch Thermal Cycler. The Visum spatial libraries were generated utilizing the Visum spatial Library construction kit (10x Genomics, PN-1000184) and sequenced on Illumina Novaseq 6000 sequencer with at least 100,000 reads per spot and paired-end reads of length 150 bp. The gene list for calculation of fibrosis score and inflammation score was based on **Supplementary Table S2**.

### Animals

The Mincle genetic (WT&KO) mice, bred on the C57BL/6J genetic background, were generously provided by Dr. Sho Yamasaki from Osaka University in Osaka, Japan (26). Male C57BL/6J mice, aged 6-8 weeks and weighing 20-25g, were obtained from Beijing Vital River Laboratory Animal Technology Co., Ltd. The mice were housed in a pathogen-free environment under a 12-hour light/dark cycle and provided with standard mouse diet and water ad libitum. All animal experiments conducted in this study received ethical approval from the Committee on the Ethics of Animal Experiments at Southeast University.

### Renal unilateral ischemic reperfusion injury model

The mice were allocated into distinct groups at random, and the same researchers carried out every surgical procedure. The abdomen was surgically opened under anesthesia to establish the UIR model. The warm renal ischemia was initiated by applying arterial clips on the left renal pedicle for 35 minutes on a 37°C-warming pad, while maintaining the integrity of the right kidney. Throughout the procedure, strict measures were taken to maintain a consistent core body temperature of mice at 36.8-37.2°C using a rectal probe for monitoring purposes. Sham-operated mice underwent identical surgical procedures, excluding the application of microaneurysm clamps. Kidney samples were collected on days 1, 3, 14, and 28 following UIR induction.

### Histopathological analysis

The kidney sections (4um) that had been fixed in formalin and embedded in paraffin were processed for periodic acid-Schiff (PAS), and Masson’s trichrome staining according to a standardized protocol. Two experienced pathologists performed the renal histopathological analysis in a blinded manner. The evaluation of renal histopathological damage included assessment of brush border loss, tubular dilation, cast formation, and tubular necrosis in 10 randomly selected tissue sections per mouse. The extent of tubular damage was evaluated using a semiquantitative scoring method to assess renal injury as a percentage: 0, no damage; 1, 10%; 2, 10–25%; 3, 25–75%; 4, >75%. Interstitial fibrosis was indicated by blue area observed in Masson staining. Renal fibrosis was quantified in at least five sections per mouse by Image J software.

### Cell experiments

A mouse macrophage cell line Raw264.7 (ATCC) was used for *in vitro* study. Raw264.7 cells were cultured in Dulbecco’s Modified Eagle Medium (DMEM; Gibco) supplemented with 1%(v/v) penicillin-streptomycin (P/S, Gibco) and 10% FBS (10099141C, Gibco). *Clec4e* knockdown in Raw264.7 cell was achieved by using lentivirus shRNA (Target sequence: CCTTTGAACTGGAAACATT) targeting the *Clec4e* gene purchased from GeneChem (Shanghai, China). Non-silencing lentivirus shRNA was used as a nonsense control (NC). Lentiviruses expressing *Clec4e* and nonsense control (NC’) constructed in the GV492 vector were purchased from Genechem (Shanghai, China). Raw264.7 cells infected with lentivirus (MOI=100) were stimulated with LPS (100ng/ml, L2630, Sigma) for 12h and then were applied for the following detection through RT-qPCR and immunofluorescence.

### Immunofluorescence staining

Prior to immuno-staining, antigen retrieval for all paraffin-embedded kidney sections was conducted using the microwave heating method in EDTA (MVS-0098, MXB Biotechnologies, Foochow, China). For immunofluorescence staining, formaldehyde-fixed kidney sections were incubated with primary antibodies against Mincle (CLEC-4E (B-7): sc-390806, Santa Cruz, USA), CD68 (ab125212, Abcam, UK), Ly6G (GB11229-100, Servicebio, CN), TNF-α (ab1973, Abcam, UK), Megalin (sc-515772,Santa Cruz, USA), α-SMA (ab5694, Abcam, UK). Raw264.7 cells seeded on the cover glasses were incubated with primary antibody against TNF-α (ab1973, Abcam, UK). Subsequently secondary antibodies were applied and DAPI was employed to stain cell nuclei. Immunostained samples were observed under a confocal microscope (FV3000, Olympus).

### Flow cytometry analysis

The Mincle-positive immune cell population in UIR kidney was quantified using flow cytometry. In brief, kidney samples were minced and enzymatically digested using the gentleMACS™ octo dissociator (Miltenyi Biotec) along with the multi tissue dissociation kit (Miltenyi, 130-110-203), followed by a 30-minute incubation at 37°C. The resulting cell suspension was filtered through 70-μm cell strainers and washed with wash buffer (PBS containing 2% FBS and 2 mM EDTA). Erythrocytes were eliminated using RBC lysis buffer (00-4333-57, eBioscience™), and cell viability was detected with Live/Dead-Fixable Viability Stain 780 (BD Biosciences, Cat. No. 565388). After blocking nonspecific Fc binding with FC Block (553141, BD Biosciences), cell suspensions were then incubated with CD45-BV510 (103138, Biolegend), CD11b-FITC (101206, Biolegend), F4/80-BV421 (565411, BD Biosciences), Ly6G-PerCP-cy5.5 (127615, Biolegend) for 30min at 4°C. For Mincle, we applied the primary anti-Mincle antibody (D266-3, MBL) and then incubated with the Alexa Fluor 647-conjunted second antibody (ab150167, abcam, UK). Flow cytometry was performed on FACSymphony A5 SORP (BD Biosciences) and data was analyzed with FlowJo software.

### Quantitative real-time PCR

The total RNA was extracted from mouse kidney samples and Raw264.7 cell lysate using RNAiso Plus (Vazyme, Nanjing, China) following the manufacturer’s protocols. Subsequent reverse transcription and quantitative real-time PCR were performed using 5× HiScript III qRT SuperMix and 2× ChamQ SYBR qPCR Master Mix (Vazyme, Nanjing, China). The expression levels of β-actin were used for data normalization, and the primers utilized in RT-qPCR were listed in **Supplementary Table S3**.

### Statistical analysis

The data were presented as the mean ± standard deviation (SD). Statistical analysis was conducted using t-test or one-way analysis of variance (ANOVA) with GraphPad Prism 9.0 software. A significance threshold of P < 0.05 indicated statistical significance.

## Results

### The expression of Mincle exhibited a biphasic pattern during the progression of renal fibrosis

**Figure 1:**
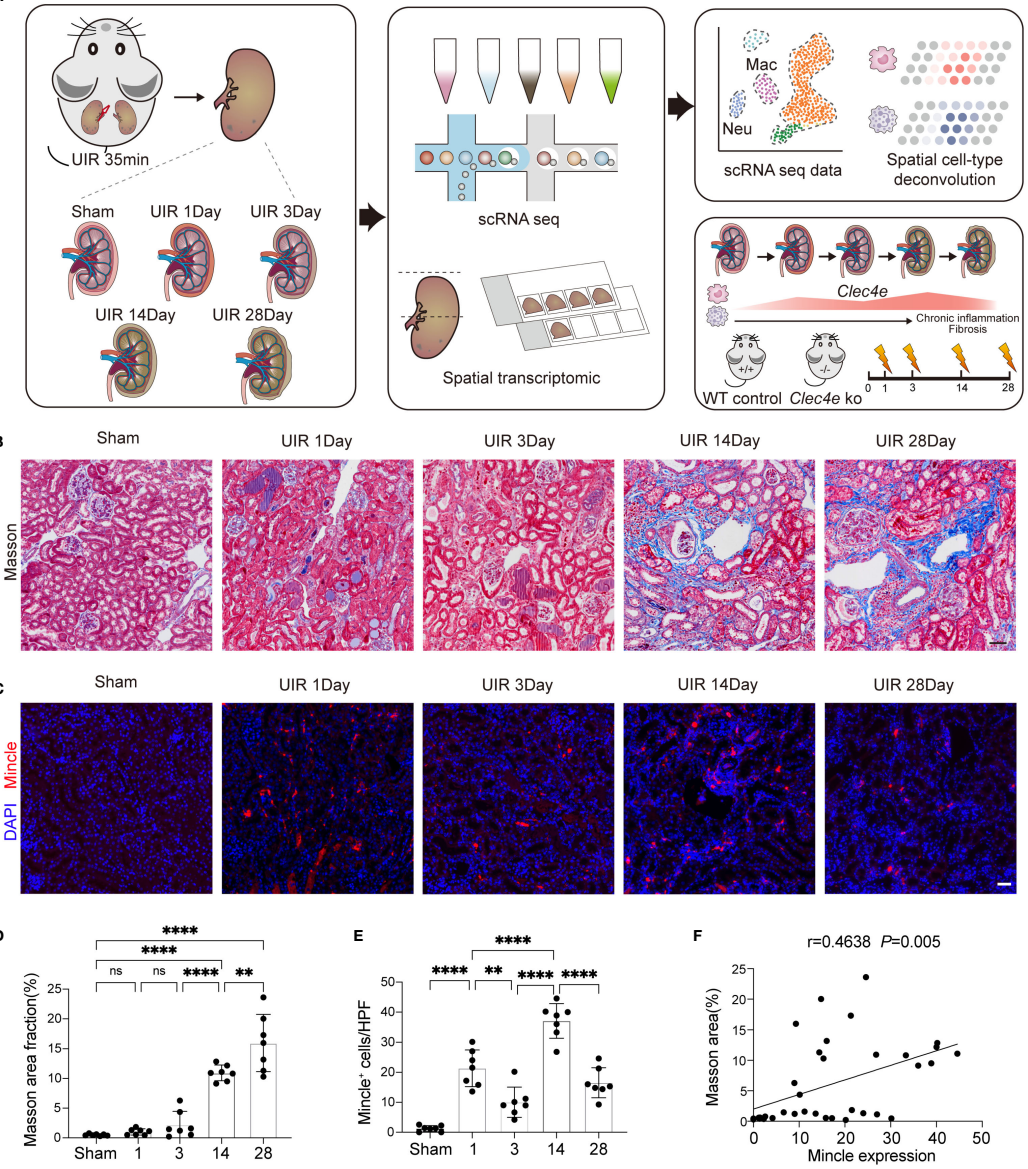
The expression of Mincle exhibited a significant correlation with kidney fibrosis. **(A)** Design and workflow of this study. The transition from AKI to CKD was experimentally induced in mice through UIR. Kidney samples collected at days 1, 3, 14, and 28 post-UIR, along with sham kidneys, were subjected to the 10x chromium single-cell and visium spatial transcriptomic procedures. Validation experiments were conducted in Mincle WT and KO mice. **(B, D)** Masson staining and quantification of kidney at various time points post-UIR. n=7. Scale bar, 50μm. **(C, E)** Representive immunofluorescencent images and quantification of Mincle (labeled in red) in kidney at various time points post-UIR. n=7. Scale bar, 50μm. **(F)** The correlation analysis between fibrosis severity, as indicated by masson area fraction, and Mincle expression was conducted. n=35. Data were presented as mean ± SD. ns, no significance, **p < 0.01, ****p < 0.0001.

To explore the dynamics and functional role of Mincle in the progression of AKI to CKD, we established a UIR-induced mouse model (**Figure 1A**). We observed remarkable renal interstitial collagen deposition on day 14, indicating the development of renal fibrosis which was aggravated on day 30 (**Figures 1B, D**). Meanwhile, a time-dependent dynamic expression of Mincle during the AKI-to-CKD transition process was noted by immunofluorescence staining (**Figures 1C, E**). We observed a biphasic pattern of Mincle expression following AKI, characterized by a sharp increase on the first day and a subsequent second peak at day 14. The Pearson correlation coefficient analysis indicated a positive association between Mincle and the extent of fibrosis suggesting potential involvement of Mincle in the chronicity of AKI (**Figure 1F**).

### The single-cell transcriptional analysis revealed the comprehensive landscape of Mincle expression in macrophages and neutrophils

**Figure 2:**
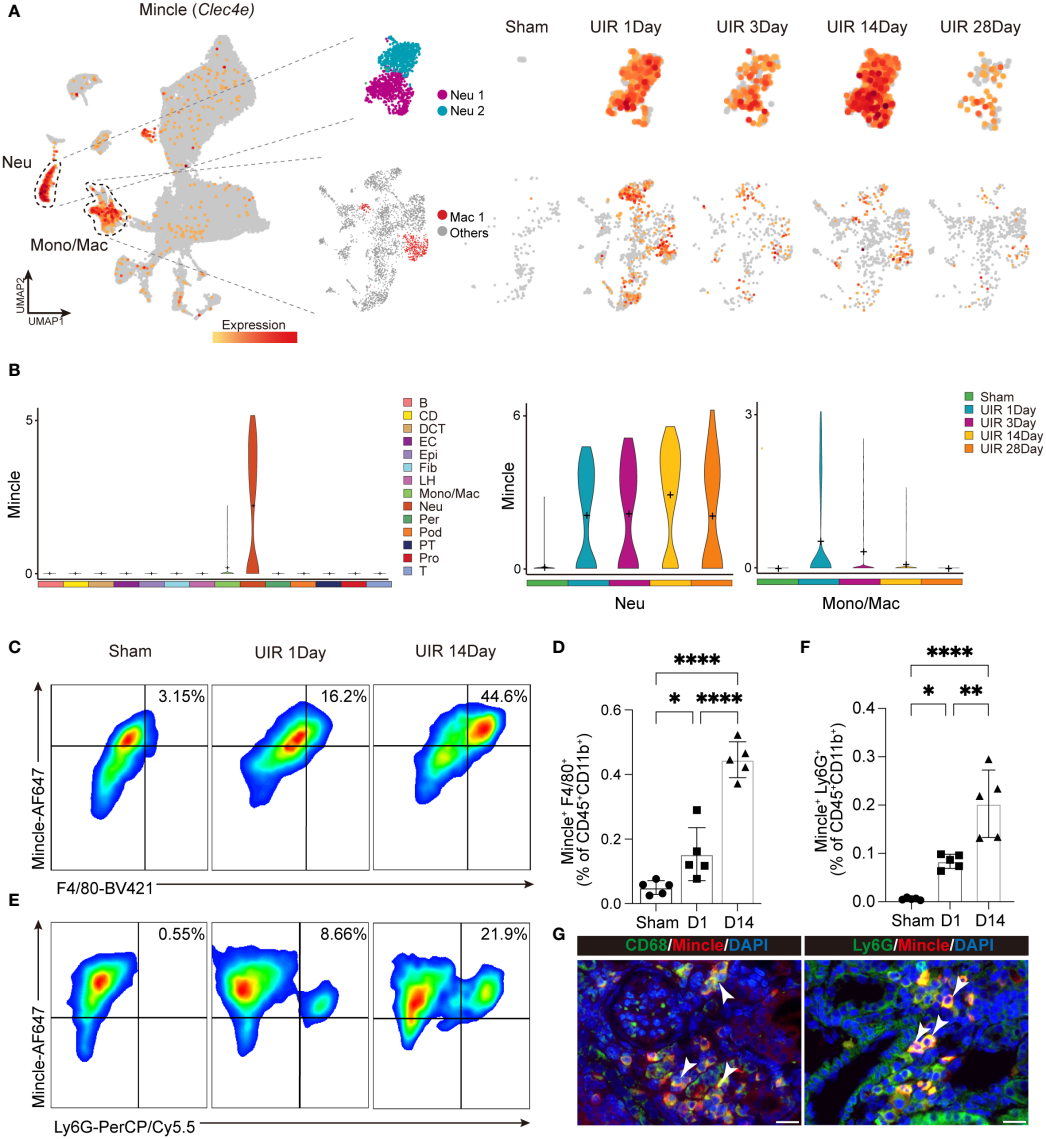
Dynamic expression of Mincle in macrophage and neutrophil. **(A, B)** The UMAP projection and violin plot suggesting expression of Mincle (*Clec4e*) in all different identified cell clusters and specific expression in neutrophil and monocyte/macrophage at different time points post injury. B, B cell; CD, collecting duct; DCT, distal convoluted tubule; EC, endothelial cell; Epi, epithelial cell; Fib, fibroblast; LH, loop of Henle; Mono/Mac, monocyte/macrophage; Neu, neutrophil; Per, pericyte; Pod, podocyte; PT, proximal tubule; Pro, proliferation cell; T, T cell. **(C–F)** Flow cytometry analysis revealing the quantification of Mincle-positive macrophages and neutrophils in the kidney from AKI to CKD (Sham, day 1 and day 14). n=5. **(G)** Representative immunofluorescence staining of CD68^+^Mincle^+^ and Ly6G^+^Mincle^+^ cells in kidney of day 14 post-UIR. CD68&Ly6G, green; Mincle, red. Scale bar, 20μm. Data were presented as mean ± SD. *p < 0.05, **p < 0.01, ****p < 0.0001.

Single-cell RNA sequencing analysis was performed to investigate the landscape of Mincle expression in cell clusters (**Figure 1A**). Our findings revealed that Mincle was mainly expressed in the populations of monocytes/macrophages and neutrophils in the kidney displaying a time-dependent pattern (**Figures 2A, B**). The Mincle expression in monocytes/macrophages and neutrophils exhibited a rapid increase on the first day post-UIR injury. However, the expression of Mincle was observed to be down-regulated in total monocytes/macrophages as disease progressed (**Figures 2A, B**), which may attribute to the decreased expression in monocyte cluster (**Supplementary Figures S1A, B**). Interestingly, unlike monocytes/macrophages, Mincle in neutrophils exhibited a sustained elevation and demonstrated a secondary peak of up-regulation on day 14 (**Figures 2A, B**). The biphasic pattern of Mincle expression on day1 and 14 suggested their critical role in the acute phase as well as the transition point towards chronicity.

To validate the findings of single-cell sequencing data, we employed flow cytometry analysis and identified a notable increase in the recruitment of Mincle-positive F4/80^+^ macrophages and neutrophils on day 1 following AKI, which further augmented on day 14 (**Figures 2C–F**). Immunofluorescence staining also displayed a large number of macrophages and neutrophils expressing Mincle in renal interstitium on day 14 (**Figure 2G**). Overall, single-cell RNA sequencing analysis unveiled a dynamic profile of Mincle expression, predominantly exhibited by macrophages and neutrophils, throughout the process of AKI-to-CKD transition.

### High Mincle-expressing neutrophils and macrophages were characterized with pro-inflammatory and pro-fibrotic signatures

**Figure 3:**
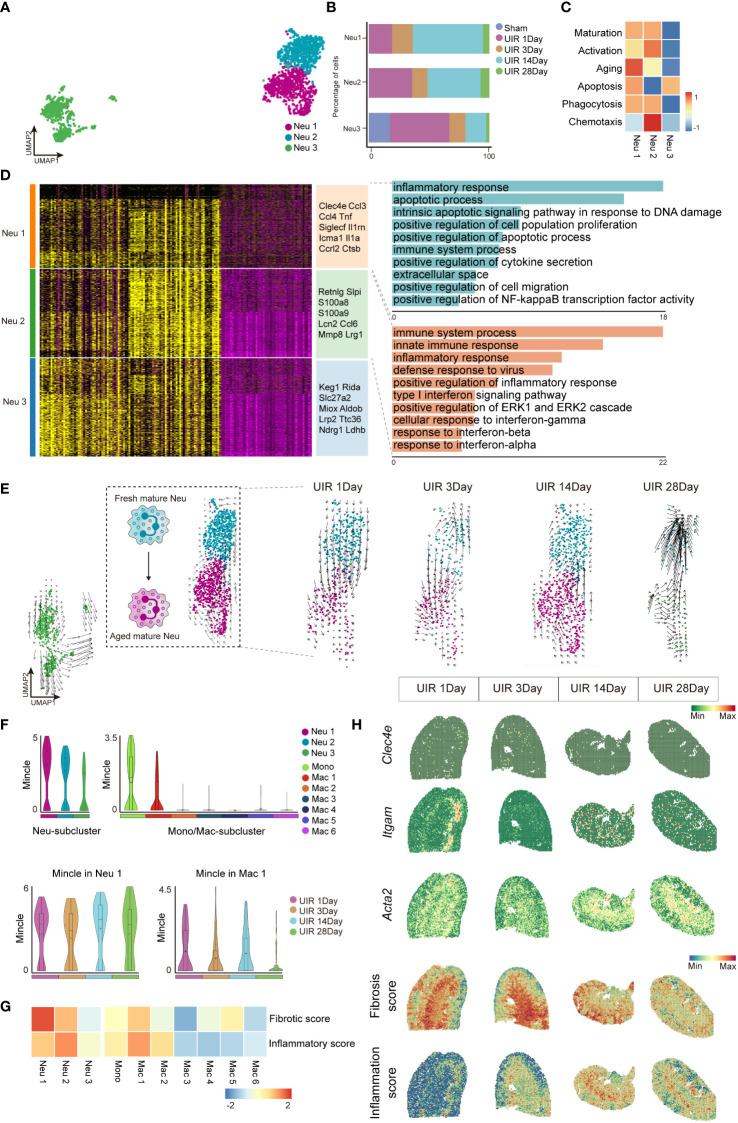
Neutrophils and macrophages expressing high levels of Mincle exhibited pro-inflammatory and pro-fibrotic signatures. **(A)** UMAP plot of all clusters of neutrophils. **(B)** The proportion of each subgroup of neutrophils at different time points. **(C)** Heatmap of phenotype and function score (including maturation, activation, aging, apoptosis, phagocytosis and chemotaxis) for each neutrophil cluster. **(D)** Heatmap of each sub-cluster of neutrophils based on DEGs and their top marker genes were listed. Top GO pathway enrichment analysis of Neu1 and Neu 2 subtypes were presented. **(E)** The UMAP plot depicting the developmental transition of neutrophil clusters in Neu 1-2 following injury, as revealed by RNA velocity analysis. **(F)** Violin plot suggesting Mincle expression in all macrophage sub-clusters and neutrophil sub-clusters, as well as the dynamics of Mincle in clusters of Neu1 and Mac1 at each time point after injury. **(G)** Heatmap of fibrotic score and inflammatory score in sub-clusters of Neu1-3 and Monocyte and Mac1-6. **(H)** Temporal and spatial gene expression patterns of *Clec4e, Itgam* and *Acta2*, along with fibrosis score and inflammation score, were elucidated using 10x Visium spatial transcriptomics.

Further analysis was subsequently conducted to delineate the characteristics of cell clusters expressing Mincle. By performing sub-clustering analysis, neutrophils were partitioned into 3 subsets displayed in UMAP plots (**Figure 3A**). The cell fraction of neutrophil cluster 1 and cluster 2 (referred to as Neu 1 and Neu 2) exhibited a significant increase on day 1, followed by obvious decline by day 3. However, with disease progression, they displayed a peak on day 14 (**Figure 3B**). Next, gene set scores were established in regard to cell maturation, activation, aging, apoptosis, phagocytosis, and chemotaxis (related genes in **Supplementary Table S2**), Neu 1 and Neu 2 were identified as mature neutrophil subsets with phagocytic function (**Figure 3C**). Neu 2 displayed early activation and chemotaxis characteristics while Neu 1 was more likely to represent an “aged” subset of neutrophils characterized with high level of *Cxcr4* and low level of *Sell* (27) (**Figure 3C**). However, Neu 3 may represent a small subset of immature or renal-resident neutrophils with undefined function (**Figure 3C**). Therefore, we focused on Neu 1 and Neu 2 in the following analysis.

Through gene ontology (GO) pathway enrichment analysis, Neu 1(high expression of *Clec4e, Ccl3, Ccl4, Tnf, Siglecf, Il1rn, Icam1, Il1a,Ccr12, Ctsb*) and Neu 2 (high expression of *Retnlg, Slpi, S100a8, S100a9, Lcn2, Ccl6, Mmp8, Lrg1*) were found to be involved in inflammatory responses (**Figure 3D**). RNA velocity analysis further elucidated the trajectory of neutrophils, revealing that Neu 2 (as a fresh mature subset) differentiated into Neu 1 (as an aged mature subset) (**Figure 3E**), which aligned well with the observed dynamic changes in neutrophil proportions as well as the defined gene set functions (**Figures 3B, C**). In addition, it turned out that Mincle was significantly up-regulated in Neu 1(**Figure 3F**). Meanwhile, unsupervised clustering identified seven distinct cell subpopulations of mononuclear phagocytes (including monocyte and Mac 1-6). Notably, a remarkable Mincle expression was observed in two specific subsets, monocyte and Mac1 (**Figure 3F**). The Neu 1 and Mac 1 clusters presenting with dual peak expression of Mincle were identified as Mincle^high^ neutrophils and Mincle^high^ macrophages, respectively. Functional assessments revealed that these Mincle^high^ neutrophils and Mincle^high^ macrophages exhibited prominent pro-inflammatory and pro-fibrotic properties (**Figure 3G**). Moreover, spatial transcriptomics data showed a pronounced expression of Mincle at the outer-stripe of outer medulla in the kidney, with similar distribution for myeloid cells (indicated by *Itgam*) as well as inflammatory regions (indicated by inflammation score) and fibrotic regions (indicated by *Acta2* expression and fibrosis score) (**Figure 3H**). Persistent chronic renal inflammation was revealed by inflammation score peaked on day 14 after AKI (**Figure 3H**).

Therefore, Mincle was highly expressed in specific subsets of neutrophils and macrophages, displaying remarkable pro-inflammatory and pro-fibrotic properties crucial for driving CKD progression following the initial kidney injury.

### Mincle^high^ neutrophils and Mincle^high^ macrophages synergistically promote the production of TNF

**Figure 4:**
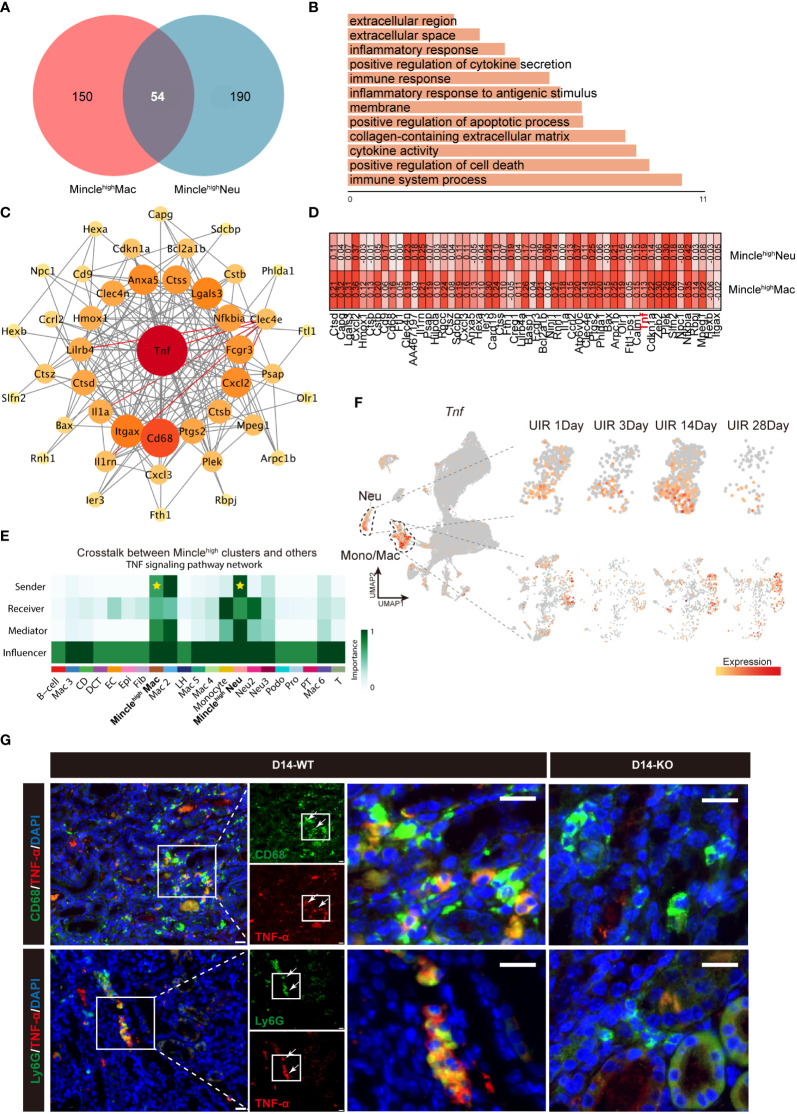
Mincle^high^ neutrophils and Mincle^high^ macrophages collaboratively facilitate TNF production. **(A, B)** DEGs and top GO terms of 54 common genes of Mincle^high^ neutrophils and Mincle^high^ macrophages. **(C)** PPI analysis of 54 common genes identified between Mincle^high^ neutrophils and Mincle^high^ macrophages. **(D)** Gene correlation analysis between *Tnf* and *Clec4e* in Mincle^high^ neutrophils and Mincle^high^ macrophages, respectively. **(E)** TNF signaling pathway indicated by relevant ligand-receptor interaction pairs predicted by CellChat between main kidney cell types. **(F)** The UMAP projection suggesting expression of *Tnf* in all different identified clusters and specific expression in neutrophils and monocytes/macrophages at different time points after injury. **(G)** Representative immunofluoresce staining of CD68^+^TNF-α^+^ and Ly6G^+^TNF-α^+^ cells in kidney of Mincle WT or KO mice on day 14 post-UIR. CD68&Ly6G, green; TNF-α, red. Scale bar, 50μm.

To further elucidate the mechanisms driving AKI-to-CKD transition mediated by Mincle-expressing macrophages and neutrophils, we identified 54 common genes from the up-regulated genes of these two cell subsets (**Figure 4A**). GO terms of these 54 genes were predicated to be implicated in immune-inflammatory responses and extracellular matrix synthesis (**Figure 4B**). In addition, protein-protein interaction (PPI) analysis showed that *Tnf* emerged as the hub gene closely related to the up-regulated genes in both Mincle^high^ neutrophils and Mincle^high^ macrophages (**Figure 4C**). Gene expression correlation analysis demonstrated a strong association between *Tnf* and *Clec4e* in these two Mincle^high^ immune cell subsets (**Figure 4D**). The CellChat algorithm-based inter-cellular communication analysis revealed important contribution of Mincle^high^ neutrophils and Mincle^high^ macrophages as ligand sources in the TNF signaling pathway network (**Figure 4E**). Correspondingly, a prominent increase in *Tnf* expression primarily in macrophages and neutrophils, particularly on day 14 was observed (**Figure 4F**). Furthermore, the *in vitro* validation confirmed a decrease in TNF expression following Mincle knockdown, while an increase was observed after overexpression of Mincle in LPS-stimulated Raw264.7 cells (**Supplementary Figures S2A–D**). Meanwhile, TNF-α expression was reduced in macrophages and neutrophils on day 14 in Mincle knockout (KO) mice (**Figure 4G**). Hence, we identified that Mincle^high^ myeloid cells (specifically macrophages and neutrophils) synergistically contributed to TNF production during the chronic transition of AKI.

### Mincle deficiency protect kidney from aggravated injury and fibrosis post-UIR

**Figure 5:**
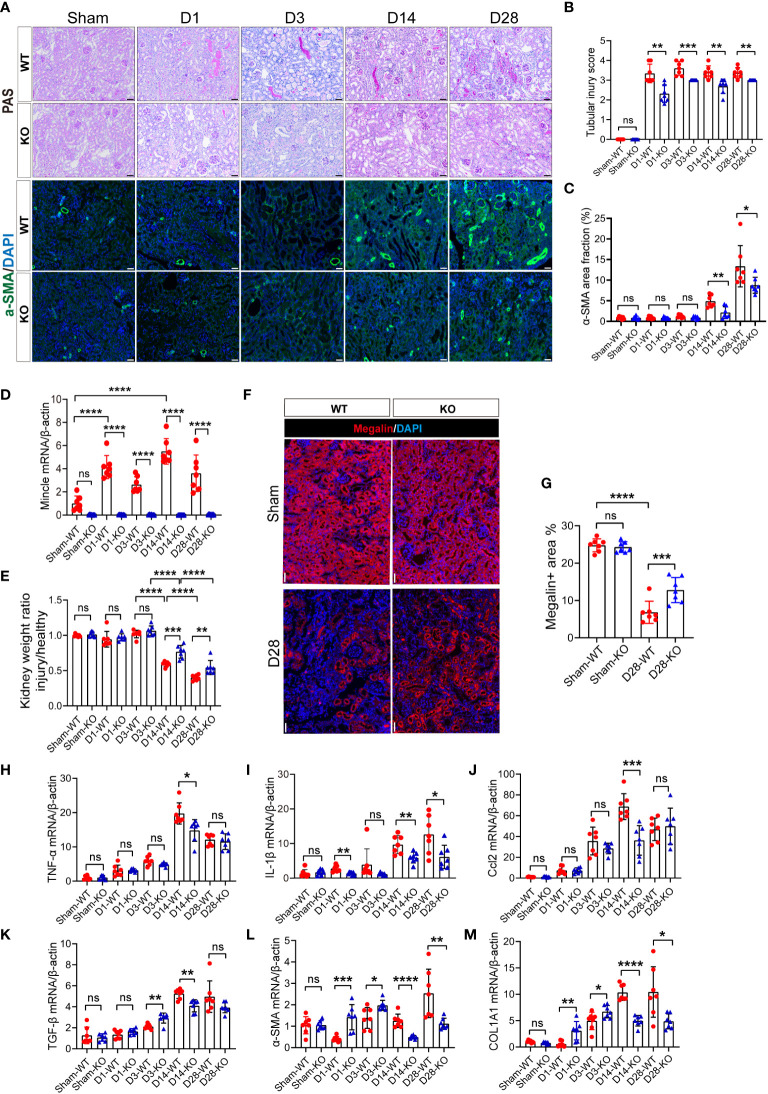
The absence of Mincle provides protection by mitigating exacerbated injury and fibrosis following UIR. **(A–C)** Acute kidney injury was induced by UIR in Mincle WT or KO mice. Renal tubular injury score was calculated according to PAS staining at each time point (Sham, day1, day3, day14 and day28) post-UIR. Representative immunofluorescent staining of α-SMA (green) in kidney at each time point after UIR. n=7. Scale bar, 50μm. **(D)** RT-qPCR analysis of Mincle mRNA in kidney from Mincle WT and KO mice. n=7. **(E)** The ratio of the weight of the injured kidney to that of the healthy (sham group) kidney. n=7. **(F–G)** Immunofluorescence staining of megalin (red) in kidney sections (Sham and day 28) after UIR with representative images. Megalin-positive areas were quantified. n=7. Scale bar, 50μm. **(H–M)** RT-qPCR analysis for the indicated inflammation and fibrosis related factors was performed on sham kidney and injured kidney samples harvested on day1, 3, 14, and 28 after injury. n=7. Data were presented as mean ± SD. ns, no significance, *p < 0.05, **p < 0.01, ***p < 0.001, ****p < 0.0001.

Due to the distinctive expression pattern of renal myeloid-derived Mincle during both early and chronic stages of kidney injury, along with its pro-inflammatory and pro-fibrotic properties, we further investigated the renal manifestations in Mincle knockout mice. In wild-type (WT) mice, obvious tubular epithelial cell (TEC) flattening, loss of the brush border and epithelial cell nuclei, and tubular cast formation were observed after AKI, which could not be completely repaired in the late stage of the disease and was accompanied by a substantial interstitial immune cell infiltration (**Figures 5A, B**). However, Mincle knockout resulted in significant amelioration in renal pathological damage at different time points during the process of AKI to CKD (**Figures 5A, B**). Additionally, α-SMA immunofluorescence staining indicated a notable attenuation of renal fibrosis in Mincle-deficient mice (**Figures 5A, C**). In Mincle WT mice, Mincle mRNA expression confirmed a biphasic up-regulation pattern following AKI (**Figure 5D**). Quantitative analysis of kidney weight and megalin staining revealed more pronounced renal atrophy and tubular loss in WT mice than those observed in KO mice (**Figures 5E–G**). Remarkably, compared to WT group, Mincle KO mice exhibited significant down-regulation in the mRNA expression of pro-inflammatory factors (TNF-α, IL-1β, Ccl2) and fibrosis-associated factors (TGF-β, α-SMA, COL1A1) especially on day 14 (**Figures 5H–M**). Collectively, our findings demonstrated that Mincle played a critical role in renal injury and fibrosis progression after AKI probably by mediating the unresolved inflammation.

## Discussion

**Figure 6:**
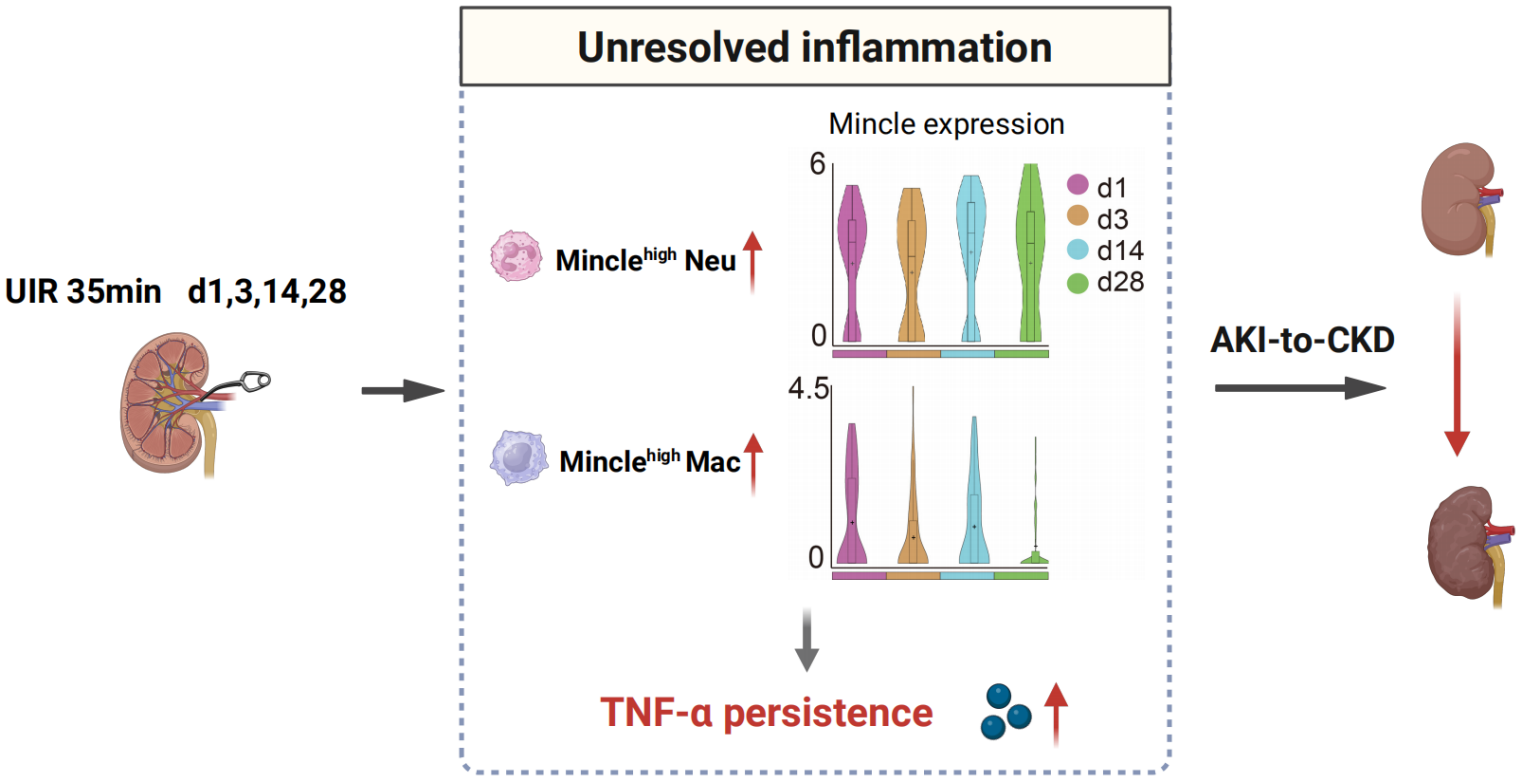
Schematic illustration depicting the involvement of Mincle in the AKI-to-CKD transition. Mincle^high^ neutrophils and macrophages contribute to unresolved inflammation by up-regulating TNF-α production, thereby driving the progression of AKI towards CKD. UIR, unilateral ischemia-reperfusion; Neu, neutrophil; Mac, macrophage; AKI, acute kidney injury; CKD, chronic kidney disease.

While Mincle has been extensively characterized as a key mediator of inflammation in AKI, further investigation is warranted to elucidate its role in the transition from AKI to CKD. In this research, we offered a comprehensive single-cell analysis of Mincle behavior during the progression from AKI to CKD. Using mouse models of UIR, we delineated the detrimental involvement of Mincle in the unresolved inflammation and subsequent renal fibrosis across the acute and chronic phase after AKI (**Figure 6**).

Initially, we identified biphasic pattern of Mincle expression during the progression of renal fibrosis which potentially contributed to the chronicity of inflammation. Previous research predominantly emphasized the pathogenic role of Mincle-expressing macrophages in AKI, confirming the initiation and exacerbation of renal inflammation by Mincle (21, 22, 28). Here, we observed a rapid up-regulation of Mincle in the initial stage of kidney injury, followed by a second peak during the chronic progression of AKI, accompanied by recruitment of immune cells and sustained inflammatory status, which exhibited a strong association with late-stage renal fibrosis. Inflammatory processes are pivotal in kidney fibrosis, involving diverse innate immune cells in establishing renal interstitial inflammation environment (2, 5). Innate immune cells not only initiate and exacerbate inflammation in the early stage, but also contribute to the progression of kidney fibrosis through sustained chronic inflammation driven by the activation of innate immune pathways (5, 7). Furthermore, in conjunction with the implementation of spatial transcriptome technology, some innate immune cells were identified in anatomical regions adjacent to fibrotic areas and were deemed to be implicated in the establishment of the renal fibrosis microenvironment (29, 30). Xu et al. recently elucidated that the sustained macrophages infiltration, along with subsequent activation of T-cells and neutrophils leading to a pro-inflammatory immune response, facilitated secondary kidney injury during AKI-to-CKD transition (11). Therefore, Mincle derived from macrophages and neutrophils may not only exacerbate early inflammatory responses but also involved in the chronic transformation of kidney injury.

Further study identified two distinct sub-clusters of Mincle^high^ macrophages and Mincle^high^ neutrophils, both exhibiting pro-inflammatory and pro-fibrotic characteristics during kidney injury progression. The recognition of functional diversity and time-dependent infiltration of immune cell signifies a critical facet in the complex pathogenesis of renal interstitial inflammation and chronic injury (7). The phenotypic heterogeneity and functional plasticity of macrophages have gained increasing interest (31). Macrophages not only participate in the early inflammatory and repair process, but also can be transformed into pro-fibrotic phenotype to mediate the collagen matrix deposition in the kidney. Utilizing single-cell RNA sequencing technology, a plethora of novel subsets of macrophages have been identified to exert distinct roles in both AKI and CKD. In the ischemia-reperfusion-induced AKI mouse model, a specific subset of monocyte-derived macrophages marked by S100a8/S100a9 expression triggered and intensified kidney inflammation (32). Additionally, an early-emerging Arg1^+^ monocyte subset displayed a pro-inflammatory and pro-fibrotic phenotype, while Ccr2^+^ macrophages appeared in late phase of injury (14). In the current study, we identified a specific Mincle^high^ macrophage population that possesses both M1 and M2 phenotypes exhibiting pro-inflammatory and pro-fibrotic characteristics, potentially sustaining renal inflammatory and fibrogenic processes. Neutrophils, recognized as primary responders in early renal injury (3, 7), have garnered increased attention in the AKI-to-CKD transition in recent researches. Persistent infiltration of specific neutrophils, such as Siglec-F^+^ or MMP-9^+^ neutrophils, is crucial for creating a pro-fibrotic microenvironment that facilitates the progressive renal fibrosis (11, 33, 34). In addition, previous report showed that the “aged” neutrophils (Cxcr4^hi^& Icam1^hi^Cxcr1^lo^) presented in the renal tissue were associated with a pro-inflammatory phenotype (35). Here, we systematically characterized three neutrophil subpopulations in UIR-induced AKI-to-CKD model. Specifically, the Mincle^high^ neutrophils were found as an “aged” sub-cluster with pro-inflammatory and pro-fibrotic signatures, prominently infiltrating during late renal UIR stages, suggesting its significant role in fibrosis progression. Therefore, Mincle^high^ macrophages and Mincle^high^ neutrophils may represent a distinct myeloid cell population implicated in inflammation and fibrosis during AKI to CKD transition. Interestingly, the expression of Mincle in neutrophils was significantly higher than that in macrophages. Nevertheless, further validation utilizing macrophage-specific Mincle-deficient mice and neutrophil-specific Mincle-deficient mice is warranted to determine whether neutrophil-derived Mincle plays a predominant role in the AKI-to-CKD progression.

Next, we found that Mincle^high^ macrophages and Mincle^high^ neutrophils contributed to the production of TNF, which was reduced significantly in Mincle knockout mice. The pathogenic role of TNF signaling pathway in kidney disease has been extensively reported. Wen et al. found that the KLF4 deficiency in myeloid cells augmented TNF-α production, thereby exacerbating necroptosis of TECs and renal interstitial fibrosis in two murine models of CKD induced by nephrotoxic serum nephritis and unilateral ureteral obstruction, however, this effect was mitigated by macrophage-specific TNF deletion (36). Utilizing single-cell transcriptomics, TNF from activated leukocytes drove Gli1^+^ cell proliferation and fostered renal fibrosis by elevating Indian Hedgehog (IHH) release from TECs (37). Moreover, an unbiased transcriptomic approach revealed shared molecular signatures of an activated kidney TNF pathway and unfavorable clinical outcomes among patients diagnosed with either minimal change disease or focal segmental glomerulosclerosis, highlighting TNF as a pivotal driver in the progression of these diseases (38). Considering the impact of TNF on renal injury and fibrosis, the dynamic infiltration of these two Mincle-expressing immune cell subsets collectively facilitates TNF production which may act as the critical contributor to the chronic transition of AKI.

Finally, we identified that Mincle deficiency protect kidney from aggravated injury and fibrosis post-UIR. New anti-inflammation therapies against renal fibrosis have been well discussed (39, 40). It has proven that targeting the endogenous ligands of Mincle represents a promising approach for alleviating renal inflammation (22, 28). Moreover, Syk inhibitors and a variety of Chinese patent medicine compounds have demonstrated the ability to suppress Mincle/Syk/NF-kB signaling pathway, consequently mitigating acute kidney inflammation induced by various etiologies (41–43). Furthermore, modulating Mincle activation via targeted manipulation of Mincle gene (23, 44) or protein receptor (45, 46) emerged as potential therapeutic avenues. Nevertheless, a limitation of this study lies in its exclusive utilization of a murine model of UIR induced AKI. Given the intricate etiologies of AKI in humans (47), it is imperative to explore alternative kidney disease models to unveil the complex mechanistic actions of Mincle in different disease contexts.

In conclusion, we have elucidated the dynamic expression of Mincle with a second peak in a UIR-mediated AKI-to-CKD model through sc-RNA seq analysis. We identified distinct Mincle^high^ macrophages and neutrophils exerting pro-inflammatory and pro-fibrotic effects, which synergistically contributed to the persistence of renal inflammatory microenvironment and accelerated renal fibrosis progression by promoting TNF production. Precisely targeting Mincle or its downstream pathways may provide a promising therapeutic avenue to impede AKI-to-CKD transition.

## Data availability statement

The data presented in the study are deposited in the GEO repository, accession number GSE267242.

## Ethics statement

The animal study was approved by The Committee on the Ethics of Animal Experiments at Southeast University (Permit No. 20210302023). The study was conducted in accordance with the local legislation and institutional requirements.

## Author contributions

CW: Writing – original draft, Visualization, Validation, Software, Resources, Methodology, Investigation, Formal analysis, Data curation, Conceptualization. YZ: Writing – review & editing, Visualization, Software, Resources, Methodology, Investigation, Formal analysis, Data curation, Conceptualization. AS: Writing – review & editing, Validation. TT: Writing – review & editing, Investigation. NL: Writing – review & editing, Software, Formal analysis. CX: Writing – review & editing, Investigation. BL: Writing – review & editing, Supervision, Project administration, Funding acquisition. LL: Writing – review & editing, Supervision, Project administration, Funding acquisition, Conceptualization.

## Supplementary material

Supplementary Figure 1 The dynamics of monocytes and Mincle-derived from the monocyte cluster. **(A)** The monocyte cluster highlighted in the UMAP projection was markedly increased on day 1 after injury. **(B)** The violin plot suggesting expression of Mincle in Mono cluster at different time points post-injury. Mono, monocyte; Mac, macrophage.

Supplementary Figure 2 Expression of TNF-α in Raw264.7 cell with Mincle knockdown or overexpression. **(A, B)** RT-qPCR analysis for Mincle and TNF-α mRNA was performed in lentivirus-infected Raw264.7 cells after stimulation with LPS. **(C, D)** Immunofluorescence staining of TNF-α (red) in Raw264.7 stimulated with LPS with representative images. The mean fluorescence intensity (MFI) of TNF-α was quantified by Image J software. n=3. Scale bar, 20μm. NC/NC’, nonsense control; KD, knockdown; OE, overexpression. Data were presented as mean ± SD. *p < 0.05, **p< 0.01, ***p < 0.001.
