# Supplementary figures and images for: Mincle receptor in macrophage and neutrophil contributes to the unresolved inflammation during the transition from acute kidney injury to chronic kidney disease
Source: Front Immunol. 2024 May 17;15:1385696. doi: 10.3389/fimmu.2024.1385696 (PMC11103384; doi:10.3389/fimmu.2024.1385696)

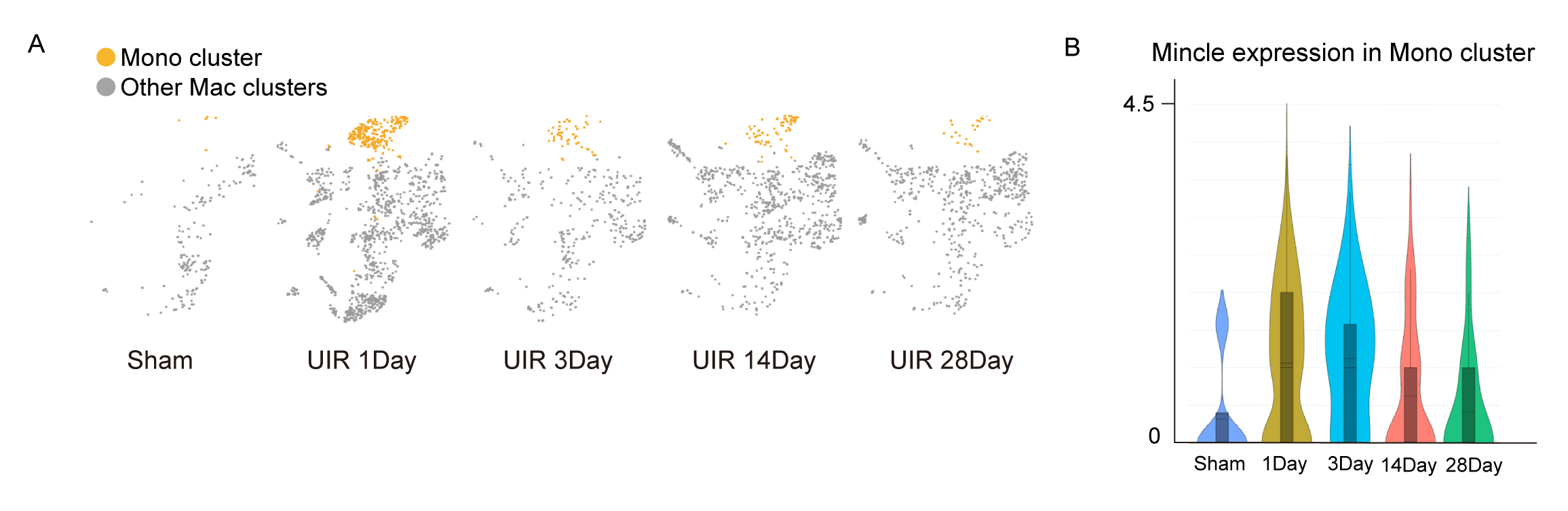

Supplement: Supplementary Figure 1 — The dynamics of monocytes and Mincle-derived from the monocyte cluster. (A) The monocyte cluster highlighted in the UMAP projection was markedly increased on day 1 after injury. (B) The violin plot suggesting expression of Mincle in Mono cluster at different time points post-injury. Mono, monocyte; Mac, macrophage. [file Image_1.tif]

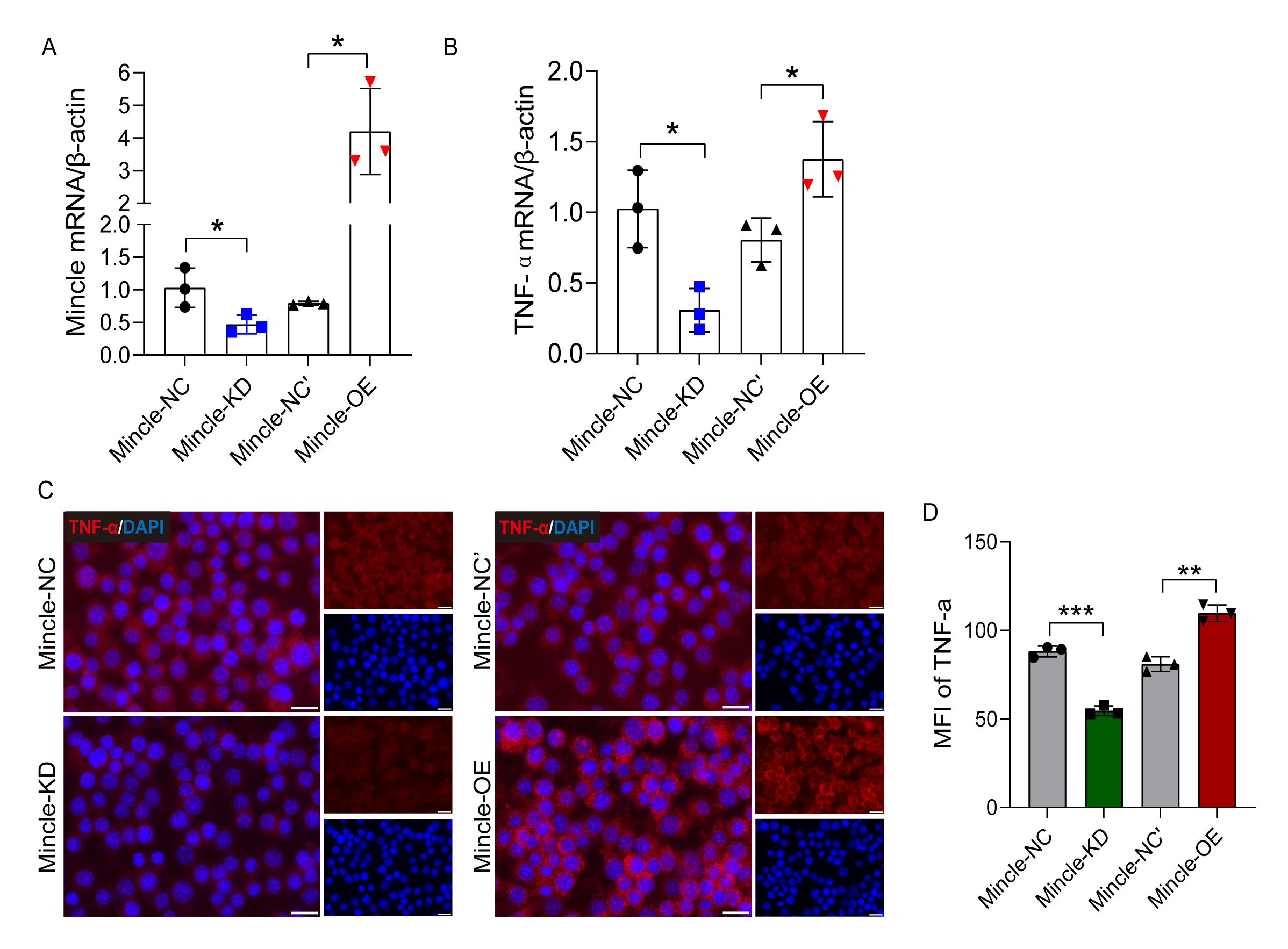

Supplement: Supplementary Figure 2 — Expression of TNF-α in Raw264.7 cell with Mincle knockdown or overexpression. (A, B) RT-qPCR analysis for Mincle and TNF-α mRNA was performed in lentivirus-infected Raw264.7 cells after stimulation with LPS. (C, D) Immunofluorescence staining of TNF-α (red) in Raw264.7 stimulated with LPS with representative images. The mean fluorescence intensity (MFI) of TNF-α was quantified by Image J software. n=3. Scale bar, 20μm. NC/NC’, nonsense control; KD, knockdown; OE, overexpression. Data were presented as mean ± SD. *p < 0.05, **p< 0.01, ***p < 0.001. [file Image_2.tif]
